# Membrane fouling induced by AHL-mediated soluble microbial product (SMP) formation by fouling-causing bacteria co-cultured with fouling-enhancing bacteria

**DOI:** 10.1038/s41598-017-09023-5

**Published:** 2017-08-16

**Authors:** So Ishizaki, Ryoichi Sugiyama, Satoshi Okabe

**Affiliations:** Division of Environmental Engineering, Faculty of Engineering, Hokkaido University, North 13, West 8, Kita-ku, Sapporo, Hokkaido, 060-8628 Japan

## Abstract

Membrane fouling still remains a major obstacle for wider applications of membrane bioreactor (MBR), which is mainly caused by soluble microbial products (SMP). Identification of key bacteria responsible for SMP production is essential for mitigation of membrane fouling. Here, we investigated the effect of microbial interaction on membrane fouling. We measured the membrane fouling potentials of 13 bacterial strains isolated from a pilot-scale MBR treating domestic wastewater when they were cultivated as single-culture and co-culture. We found that fouling-causing bacteria (FCB) displayed much higher fouling potential when co-cultured even with non-FCB and mixed population (activated sludge). In particular, the fouling potential of strain S26, one of FCB, increased 26.8 times when cultivated with strain S22 (fouling-enhancing bacteria, FEB). The secretion of *N*-octanoyl-L-homoserine lactone (C8-HSL) was increased by co-cultivating S22 and S26 as compared with cultivating as single culture, which stimulated the production of fouling-causing SMP by S26 and consequently resulted in severe membrane fouling. This result suggests that AHL-mediated quorum-sensing (QS) regulatory system was involved in secretion of fouling-causing SMP.

## Introduction

Membrane bioreactor (MBR) is considered to be a core wastewater reclamation technology to fulfill our diverse water demand^1, 2^. However, membrane fouling is still one of the main obstacles for its wider applications^2, 3^. Soluble microbial product (SMP) and extracellular polymeric substances (EPS) were considered to be main causes of membrane fouling in MBRs^4^. Therefore, better understanding of the detailed mechanisms of SMP and EPS production is essential for membrane fouling mitigation and control strategies^5^.

Extensive studies have been conducted to isolate and characterize key bacteria responsible for membrane fouling^6–9^. In our previous study, forty-one bacterial strains were isolated from a pilot-scale MBR treating municipal wastewater, tested for their fouling potential using a cross-flow filtration unit, and related to their cellular properties^8^. Thirteen out of 41 isolated strains displayed significant fouling potentials when they were cultivated as single-cultures, and thus were considered as fouling-causing bacteria (FCB). However, since bacteria were commonly present as mixed species in actual MBRs, the influence of microbial interaction of isolated bacterial strains on membrane fouling should be examined.

Several studies have revealed microbial interactions stimulated the formation of thicker biocake^10, 11^. The bacteria initially colonized on membrane surface and their SMP and EPS secretion facilitated subsequent bacterial attachment and growth, and eventually caused severe membrane fouling^10, 11^. However, it is not clear how microbial interaction influences the production of SMP and EPS and consequently membrane fouling.

It is well known that a variety of bacteria are capable of producing signal molecules (i.e. *N*-acyl homoserine lactones (AHL)), communicating each other and regulating gene expression in response to population density, which is known as AHL-mediated quorum-sensing (QS) system^12^. Several AHLs-producing bacteria were identified in MBRs^13^. Furthermore, several studies have revealed the links between the presence of various AHL signals with biocake formation and membrane fouling in MBRs^14–16^. Although the amounts of SMP and EPS in both biocake and mixed liquor were related to the increase in AHL concentrations^15, 16^, the exact role and mechanism of AHL-based QS in membrane fouling are not still clearly understood due to complex microbial interactions.

The aim of this study is, therefore, to investigate whether microbial interaction influences on the fouling potential. To achieve this goal, thirteen bacterial strains isolated in our previous study were tested for their fouling potential when cultivated as single-culture and co-culture. As a result, three strains exhibited significant fouling potential as single-cultures, thus they were defined as fouling-causing bacteria (FCB). Furthermore, co-culturing strain S26, one of the FCB, with strain S22 dramatically increased the fouling potential, which was caused by AHL-mediated SMP production by S26. The SMP produced in the co-culture of FCB and fouling-enhancing bacteria (FEB, S22 in this study) was further characterized.

## Materials and Methods

### Bacterial strains

Representative strains were selected from 17 operational taxonomic units (OTUs) composed of 41 bacterial strains isolated in our previous study^8^ (Table 1). However, since 4 strains could not be cultured on M9 medium, only 13 strains were used for the following studies. The degree of fouling potential of the isolated strains was categorized in three groups (high, I; moderate, II; low: III)^8^ (Table 1). All the strains were cultivated with M9 medium containing 48 mM Na_2_HPO_4_, 22 mM KH_2_PO_4_, 8.5 mM NaCl, 19 mM NH_4_Cl, 2 mM MgSO_4_, 100 μM CaCl_2_, 20 mM glucose, and trace element.

**Table 1:** Bacterial strains used in this study.

Strain	Closest species	Sequence similarity (%)	^a^Fouling potential
S01	*Escherichia coli*	99.9	III
S05	*Klebsiella pneumoniae*	99.5	I
S09	*Enterobacter aerogenes*	98.6	III
S12	*Leclercia adecarboxylata*	97.1	II
S14	*Pseudomonas aeruginosa*	99.9	III
S15	*Pseudomonas alkylphenolia*	99.3	II
S18	*Acidovorax delafieldii*	98.8	III
S20	*Pseudoxanthomonas mexicana*	99.8	II
S22	*Thermomonas fusca*	98.5	II
S26	*Mesorhizobium ciceri*	98.4	I
S31	*Bacillus subtilis*	100.0	III
S32	*Paenibacillus polymyxa*	99.4	I
S40	*Microbacterium azadirachtae*	99.6	I

^a^Fouling potential: The degrees of fouling potential of the isolated strains were determined with cross-flow membrane filtration system (CFMFS) when cultivated as single-culture and categorized as follow: high, I; moderate, II; low: III^8^.

### Measurement of membrane fouling potential

Membrane fouling potential of isolated strains was determined by dead-end filtration. Colonies of each individual strain grown on R2A agar plates was suspended in M9 medium, and OD_600_ of the bacterial suspension was adjusted to 1.0 with fresh M9 medium. For single-culture studies, 0.5 mL of the bacterial culture (OD_600_ = 1.0) were inoculated into 50 mL of M9 medium and incubated for 48 hr at 30 °C. For co-culture studies, two strain cultures (0.25 mL each, OD_600_ = 1.0) were inoculated into 50 mL of M9 medium and incubated for 48 hr at 30 °C. For mixed population (sludge) study, each culture (0.05 mL, OD_600_ = 1.0) of FCB and mixed liquor (0.45 mL, OD_600_ = 1.0) of MBR treating municipal wastewater was inoculated into 50 mL of M9 medium and incubated for 48 hr at 30 °C^17^. After incubation, OD_600_ values of all the bacterial cultures were adjusted to approximately 0.3 with fresh M9 medium to eliminate cell concentration.

The supernatant after centrifugation (at 4 °C, 6,000 × *g* for 15 min) of the bacterial culture was subjected to dead-end filtration test to evaluate the fouling potential. Five mL of the supernatant was transferred to a filtration unit (UHP-25K; Advantec Toyo; Tokyo, Japan) with a flat membrane filter (Pore size is 0.2 μm and membrane diameter is 25 mm, hydrophilic PTFE; Advantec Toyo; Tokyo, Japan), and filtered with continuous stirring under 50 kPa of ambient air. Thereafter, MilliQ water (approximately 10 mL) was added in the filtration unit and filtered again under the same pressure but without continuous stirring^18^. The permeate flow rate of MilliQ water was measured, and the membrane resistance of the fouled membrane was calculated as follows;

$${\rm{Membrane}}\,{\rm{resistance}}\,({{\rm{m}}}^{-1})=\mathrm{PA}/{\rm{\mu }}{\rm{Q}}$$

where, P is the pressure (Pa), A is the filtration area of membrane (m^2^), μ is the dynamic viscosity of MilliQ water (Pa · s), and Q is the permeate flow rate of MilliQ water (m^3^/s).

### Effect of supernatant and AHL on fouling potential

To investigate the microbial interaction between fouling-causing bacteria (FCB) and fouling-enhancing bacteria (FEB), S26 was cultured with the supernatant of either S22 or S31, and its fouling potential was measured. For the cultivation of S26, M9 medium was made with the supernatant of bacterial culture (OD_600_ = 0.3) sterilized with syringe filter (0.2 μm, Mixed Cellulose Ester; Advantec Toyo; Tokyo, Japan) to check the effect of microbial interaction on fouling potential via bacterial secretion. S22 and S31 were also cultured with M9 medium made with the supernatant of S26 to confirm the effect of the supernatant of FCB.

In order to evaluate the effect of AHL on enhancement of fouling potential, S22 and S26 was cultivated with M9 medium containing 4.4 μM of *N*-octanoyl-L-homoserine lactone (C8-HSL). The bacterial cultivation and the fouling potential measurement were carried out as mentioned above.

### Thin-layer chromatograph (TLC) assay for AHL

TLC assay was conducted to identify the AHL produced by isolated strains as described elsewhere in previous study with small modification^19, 20^. Bacterial cultures of S22 and S26 as single culture, and S22 and S26 as co-culture culture were prepared as described for measurement of membrane fouling potential. After 48 hr incubation at 30 °C, the bacterial cultures was centrifuged (4 °C, 6,000 × g, 15 min) and filtered with the sterilized syringe filter (0.45 μm, Mixed Cellulose Ester; Advantec Toyo; Tokyo, Japan) to remove bacterial cells. The supernatant (100 mL) was concentrated about 20 times by freeze-dry and AHL was extracted four times with 10 mL of ethyl acetate. The ethyl acetate was evaporated and the residual was dissolved with 10 μL of ethyl acetate. This solution and 5 μL of AHL standards were spotted on the C18 reversed-phase plate (TLC silica gel 60 RP-18 F254s; Merck Co.; Darmstadt, Germany) and the chromatogram was developed with methanol/water (60:40, v/v). The production of AHL was determined with *Chromobacterium violaceum* VIR24 (VIR24)^21^. After dried in air, the plate was overlaid with thin film of the mixture of LB broth containing 0.6% (w/v) and an overnight culture of VIR24 at the ratio of 1:1. After the incubation overnight at 30 °C, purple spot appeared in response to the presence of AHL on the plate. Based on the color development, R_f_ value of AHL standard and AHL produced by the strains were estimated as described elsewhere^16^. Each spot was analyzed by using ImageJ to quantify the concentration of AHL using commercial C8-HSL (Cayman Chemical Co.; MI, USA) as the standard^22^.

### SMP extraction and characterization

In this study, SMP was defined as total organic carbon (TOC) in the supernatant after centrifugation of bacterial culture at 4 °C, 6,000 × *g* for 15 min. The supernatant was dialyzed to remove residual glucose as described elsewhere^23^. OD_600_ value of all the culture was adjusted to approximately 0.3 with fresh M9 medium to eliminate cell concentration effect before the dialysis. Carbohydrate and protein concentrations in dialyzed SMP were quantified with phenol-sulfonic acid method using glucose as the standard and Lowry method using BSA as the standard, respectively. In order to estimate the quantities of SMP trapped on membrane, carbohydrate and protein in SMP before and after the dead-end filtration was measured^24^.

SMP was also characterized by using fourier transform infrared (FTIR). The spectrum of the freeze-dried SMP was measured by a FTIR spectrometry (FT/IR-660 Plus; JASCO Co.; Tokyo, Japan). The operating range was from 4000 to 1000 cm^−1^ with a resolution of 2 cm^−1^, and transmittance spectra were generated^25^. Principle compartment analysis (PCA) was carried out with the normalized spectra^26^.

All the statistical analyses including PCA were carried out with R 3.0.2 (R Development Core Team; Vienna, Austria). *P* values less than 0.05 were considered statistically significant in all analyses.

## Results

### Fouling potential of isolated strains as single-culture

**Figure 1:**
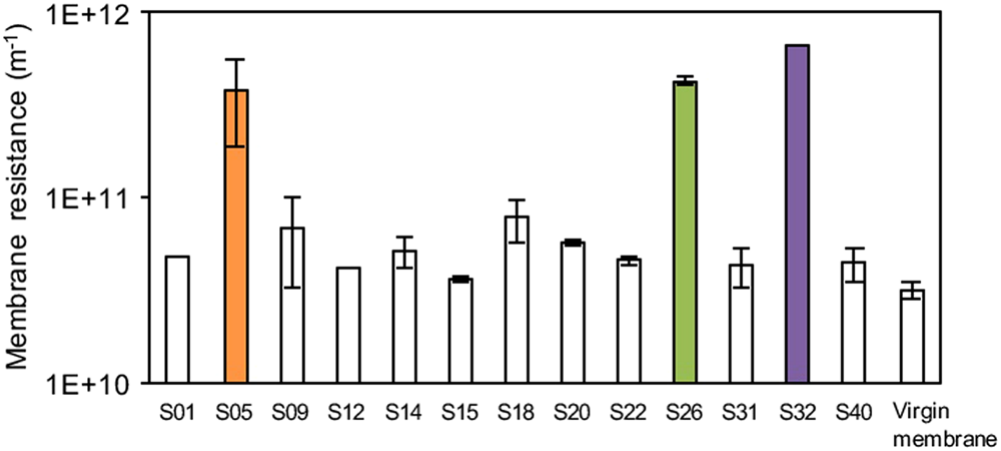
Fouling potential of 13 isolated strains (see Table 1) cultivated as single-cultures and virgin membrane. Since S05, S26, and S32 exhibited high fouling potential, they were regarded as fouling-causing bacteria (FCB) in this study. The error bars indicate the standard deviations of two independent experiments.

Fouling potential of 13 isolated strains cultivated as single-cultures was determined by dead-end filtration (Fig. 1). Strain S05, S26, and S32 showed significantly high fouling potential as compared with other strains. Therefore, these strains were considered as fouling-causing bacteria (FCB) in this study.

### Fouling potential of isolated strains as co-culture

**Figure 2:**
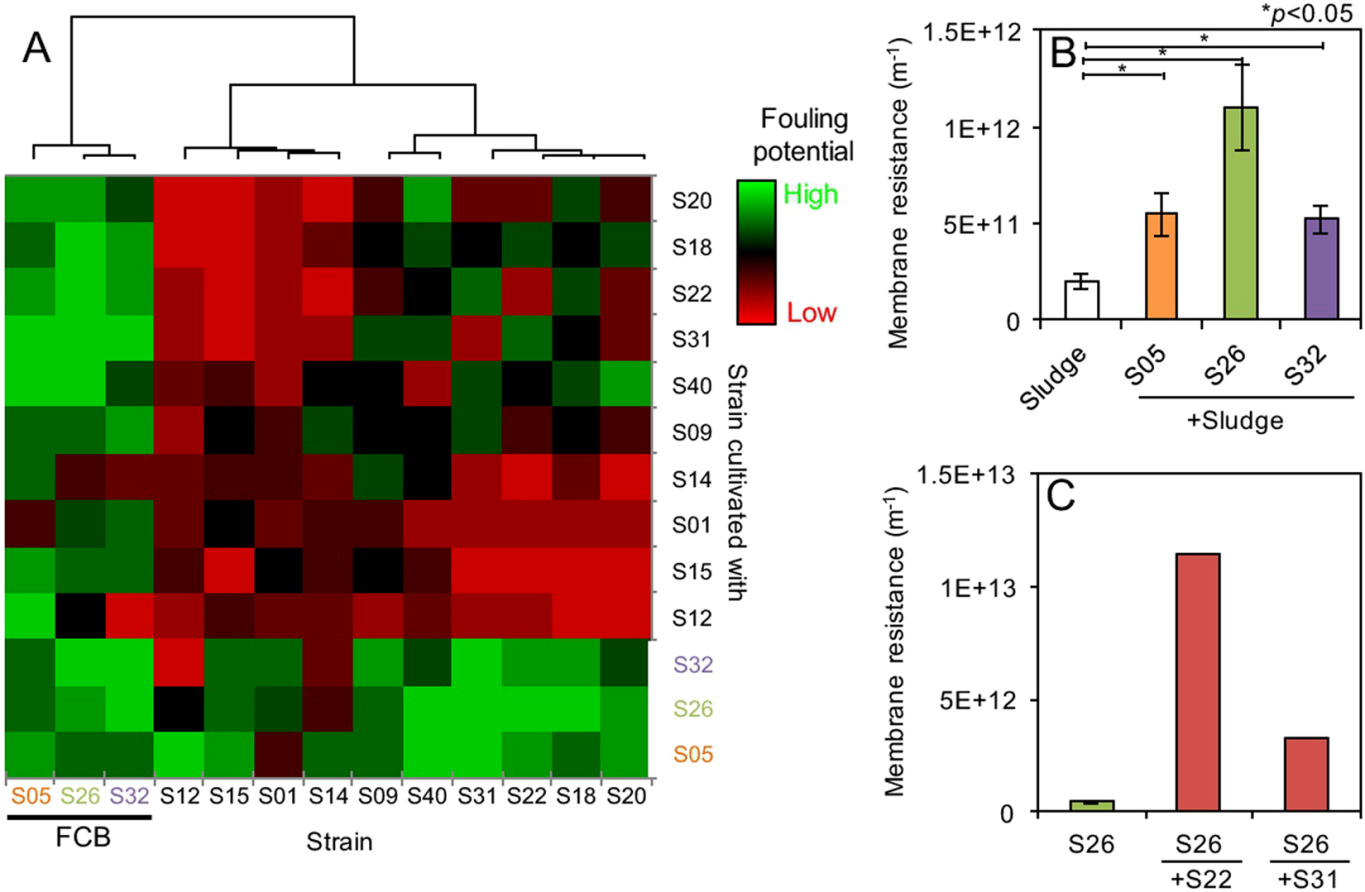
(**A**) Fouling potential of co-culture of 13 isolated strains (in total 78 combinations). (**B**) Effect of FCB (S05, S26, and S32) addition to activated sludge on fouling potential. The error bars indicate the standard deviations of two independent experiments. The bar following sign indicates statistical difference between two groups with respective *p* value: *P < 0.05. (**C**) Fouling potential of S26 as single-culture and co-culture with S22 or S31.

Fouling potential of 13 isolated strains was determined by dead-end filtration when they were cultivated as co-culture (Fig. 2A). There were 78 possible co-culture combinations of 13 isolated strains (the fouling potential and the OD600 value of all combinations were summarized in Tables S1 and S2, respectively). The fouling potential was elevated when all strains except for a few strains (S01, S12, and S14) was co-cultured with FCB (S05, S26, and S32). On the other hand, co-culturing with non-FCB did not significantly promote the fouling potential (Fig. 2A). These results suggest that membrane fouling was mainly caused by FCB, and some strains (S01, S12, and S14) might have capability to mitigate membrane fouling of FCB.

Furthermore, FCB were mixed with activated sludge of MBR treating municipal wastewater and cultured for 2 days at 30 °C, and then the fouling potential of culture medium was measured (Fig. 2B). The addition of all FCB significantly enhanced the fouling potential of activated sludge (*p* < 0.05), suggesting that FCB dominantly responsible for membrane fouling even in complex mixed populations.

### Significant increase in fouling potential by co-culturing S22 and S26

**Figure 3:**
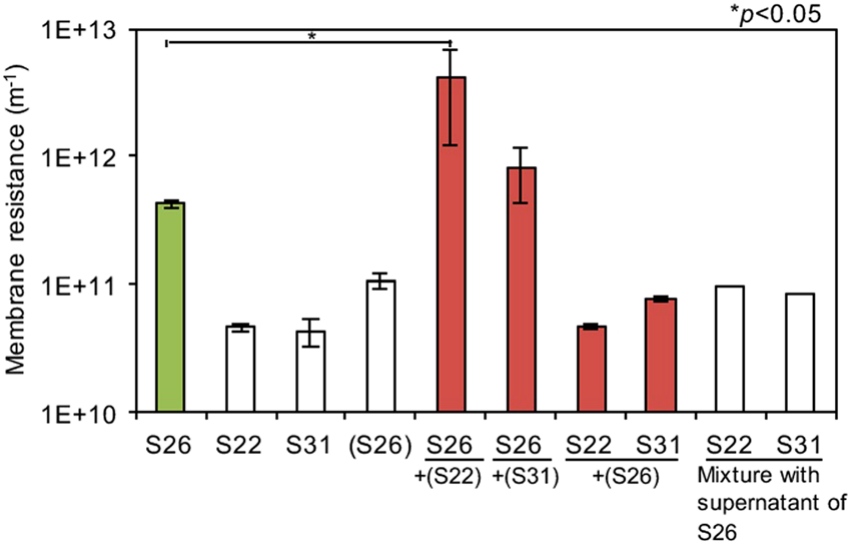
Effect of addition of the supernatants on fouling potential. The filter-sterilized supernatant of bacterial culture was added to M9 medium for cultivation of other strains. For example, S26 + (S22) indicates that S26 was cultured in the M9 medium with the filter-sterilized supernatant of S22 culture. The mixture of supernatants was also tested for fouling potential to assess abiotic effects by co-cultivating. The error bars indicate the standard deviations of two replicates except for the mixture of supernatants. The bar following sign indicates statistical difference between two groups with respective *p* value: *P < 0.05.

When S26 was co-cultured with S22 or S31, the fouling potential increased to 1.1 × 10^13^ and 3.3 × 10^12^ (m^−1^), respectively, which were 26.8 or 7.8 times greater than the one of S26 single-culture (4.3 × 10^11^ (m^−1^)) (Table S1, Fig. 2C). The growth of S26 was similar regardless of single and co-cultures (Fig. S1). Furthermore, the fouling potential of S26 was significantly increased when S26 was cultured in M9 medium made with the filter-sterilized supernatant of S22 culture medium (9.6 times, *p* < 0.05) (Fig. 3). On the other hand, the fouling potential of S22 and S31 did not change when S22 and S31 were cultured in the M9 medium made with the filter-sterilized supernatant of S26 culture medium (Fig. 3). In addition, the fouling potential of the supernatant S26 did not change when mixed with the supernatant of S22 or S31, indicating that abiotic interaction such as aggregation did not affect the membrane fouling (Fig. 3). These results suggest that production and secretion of fouling-causing matter, probably soluble microbial product (SMP), by S26 was enhanced by addition of the filter-sterilized supernatant of S22 or S31 culture medium. Thus, S22 and S31 were considered as fouling-enhancing bacteria (FEB) in this manuscript.

The strain S22 was affiliated with the Genus *Thermomonas* and shared 98.5% of 16 S rRNA gene sequence with *Thermomonas fusca* DSM 15424^27^ (Table 1). The S26 was affiliated with the Genus *Mesorhizobium* and shared 98.4% of 16 S rRNA gene sequence with *Mesorhizobium ciceri* biovar biserrulae WSM1271^28^ (Table 1). The S31 was closely related to *Bacillus subtilis* (100%), which is a gram negative bacteria. *Mesorhizobium* is known to produce AHL^29^. Although the ability of *Thermomonas* to produce AHL is not reported in previous studies, the genus *Stenotrophomonas*, which is also affiliated with the Family *Xanthomonadaceae* like *Thermomonas*, is known to produce AHL^30^. In addition, *Mesorhizobium* is known to possess LuxR/LuxI-type quorum-sensing regulatory system^31, 32^. Based on these evidences, it is speculated that the increased fouling potential of S26 is related to AHL-mediated processes.

**Figure 4:**
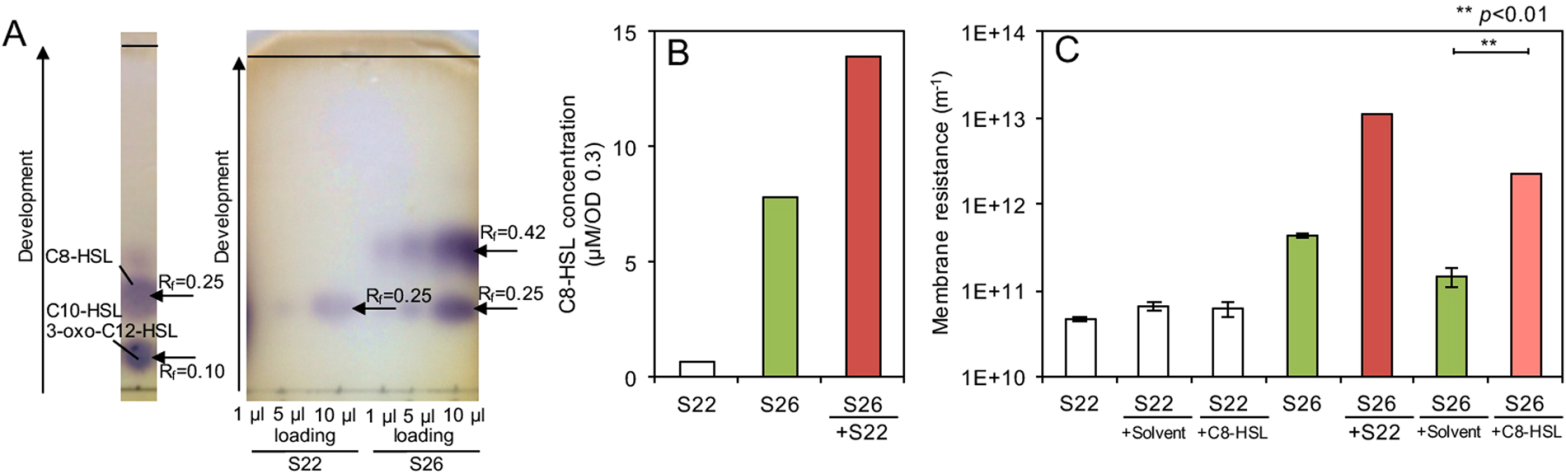
(**A**) Thin-layer chromatograph assay of *N*-acyl-homoserine-lactone (AHL) produced by S22 and S26 (Full-size images were shown in Fig. S2). (**B**) *N*-octanoyl-L-homoserine lactone (C8-HSL) concentration produced by single-culture of S22, S26, and co-culture of S22 and S26. (**C**) Effect of C8-HSL addition on the fouling potential of S22 and S26 culture. The error bars indicate the standard deviations of two independent experiments. The bar following sign indicates statistical difference between two groups with respective *p* value: **P < 0.01.

TLC assay was performed to determine the production of AHL by S22 and S26. As a result, it was confirmed that both S22 and S26 produced most likely *N*-octanoyl-L-homoserine lactone (C8-HSL), but S26 produced more significantly than S22 (Fig. 4A and Fig. S2). In contrast, the productions of any AHL by the other FCB (i.e., S05 and S32) and S31 were not confirmed (data not shown). The C8-HSL production by S26 was increased by 78% when co-cultured with S22 (Fig. 4B). Furthermore, the fouling potential of S26 was significantly enhanced when S26 was co-cultured with S22 and cultured in M9 medium containing 4.4 μM C8-HSL for 2 day at 30 °C (*p* < 0.01) (Fig. 4C). In contrast, addition of C8-HSL did not increase the fouling potential of S22 (Fig. 4C). These results indicated that the increased production of C8-HSL during co-culturing S26 with S22 may have caused the increased fouling potential of S26.

### Characterization of SMP

**Figure 5:**
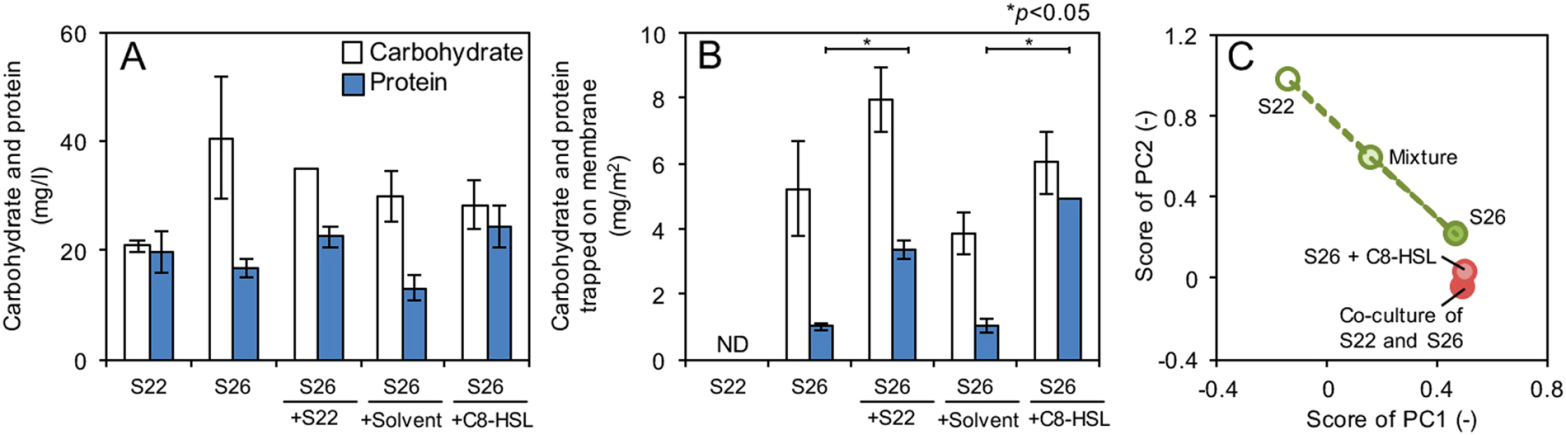
(**A**) Carbohydrate and protein concentrations in soluble microbial product (SMP) produced by S22, S26, S22 + S26 (co-culture) and S26 cultured with C8-HSL (4.4 μM). (**B**) Carbohydrate and protein contents in SMP trapped on membrane surface in the dead-end filtration experiment for these cultures. The error bars indicate the standard deviations of two independent experiments. The bar following sign indicates statistical difference between two groups with respective *p* value: *P < 0.05. (**C**) Principal compartment analysis based on fourier transform infrared (FTIR) spectrum of SMP. “Mixture” indicates the mixture of the SMP produced by strain S22 and S26 with a ratio 1:1. The SMP produced by co-culture was close to the SMP produced by S26, indicating that the co-culture SMP was mostly produced by strain S26.

The carbohydrate content in SMP produced by S26 was higher than that of S22 (Fig. 5A), which might reflect the difference of fouling potential (Fig. 4C). Although total amount of SMP (carbohydrate and protein) did not change when S26 was cultured with S22 and C8-HSL (Fig. 5A), the membrane fouling potential was significantly increased (Fig. 4C). However, amount of SMP trapped on the membrane surface after filtration was significant different. The more SMP was detected on the membrane surface when strain S26 was cultured with S22 and C8-HSL (Fig. 5B), which consisted with the increase in the fouling potential (Fig. 4C). In contrast, no accumulation of SMP was found for the S22 culture medium. These results might indicate that the composition or characteristics, not total quantity, of SMP was an important factor determining membrane fouling and changed through C8-HSL produced by co-culturing with S22.

The composition of SMP was characterized with PCA based on the spectrum gained by a FTIR spectrometry (Fig. 5C; the FTIR spectra were shown in Fig. S3). The FTIR spectrum of SMP in the co-culture of S26 and S22 was different from that in the single-culture of S22 and S26. For example, peaks around 3300 cm^−1^ and 1080 cm^−1^ in the co-culture or S26 + C8-HSL were bigger than in both single-cultures. There were more peaks in the range of 2500–1500 cm^−1^ in the co-culture than both single-cultures. The peaks around 3300 and 1080 cm^−1^ were designated as the presence of carbohydrate-like, while the peaks around 1650 cm^−1^ were designated as protein-like substances^17, 25, 33^. The SMPs produced by a single-culture S22 and S26 were plotted apart from each other, indicating different compositions. The mixture of the supernatants of single-culture S22 and S26 (1:1) was located in the middle of both plots (Fig. 5C). However, the plot representing co-culture of S22 and S26 was located near the plot representing S26 rather than S22, suggesting the co-culture SMP was resemble the SMP produced by S26 (Fig. 5C). Moreover, the SMP produced by S26 with exogenous C8-HSL was close to the plot representing co-culture of S22 and S26, suggesting that the composition of SMP produced by the co-culture was more similar to that by S26 under the presence of exogenous C8-HSL.

## Discussion

In our previous study, the fouling potential of 41 phylogenetically distant strains that were previously isolated from the fouled membranes of a pilot-scale MBR treating real domestic wastewater were evaluated as single-culture^8^. However, the effect of co-culturing these isolates on membrane fouling was unknown presently; therefore it was investigated in the present study. It was found that FCB induced severe membrane fouling even in co-cultures with non-FCB and complex microbial community (activated sludge) (Fig. 2). In particular, the fouling potential of S26, one of the FCB, was increased 26.8 times when cultivated with S22 that stimulated the production of fouling-causing SMP by S26. On the other hand, any co-culture of non-FCB did not show higher fouling potential than those of single FCB cultures (Table S1). It is, therefore, speculated that FCB could be responsible for membrane fouling in the pilot-scale MBRs treating domestic wastewater, suggesting that membrane fouling potential of single-cultures of isolated strains provided useful, but limited, information.

The microbial interaction that stimulated fouling-causing SMP production by S26 and consequently caused severe membrane fouling was further investigated in detail. The production of SMP was considered to be highly dependent on microbial populations in MBR^34^. However, our findings demonstrated the secretion of C8-HSL molecule was significantly increased by co-culturing S26 and S22, which induced S26 to produce fouling-causing SMP (change the SMP composition), leading to severe membrane fouling (Fig. 5). Therefore, the concentration of C8-HSL was one of key factors to determine the fouling potential of co-culture of S26 and S22. When S26 was co-cultured with S31, the fouling potential was 7.8 times greater than the one of S26 single-culture. However, S31 was closely related to a gram negative bacterium, *Bacillus subtilis* (100%), which did not produce AHL, suggesting other regulatory mechanism could be involved in the increased fouling potential of S26.

Although many studies have examined the role of AHL-mediated QS in pure culture systems so far, available reports for co-culture or defined population systems are very limited. Given extensive studies so far, it is now conceivable that AHL-mediated QS system is important for biofilm formation. It is also known that EPS synthesis is subject to AHL molecules in both qualitative and quantitative manner^35–37^. It was reported that the AHL-mediated QS was involved in production and secretion of hydrophobic extracellular proteins, which promote microbial aggregation of activated sludge^38^. The carbohydrate and protein contents in EPS were also regulated by AHL^38, 39^. In MBRs, it was reported that the amounts of SMP and EPS in both biocake and mixed liquor were closely related to the increase in AHL concentration^16^. However, the exact mechanism of AHL in membrane fouling was not clearly revealed in these studies. To the best of our knowledge, the present study showed for the first time that AHL concentration was elevated by co-culturing FCB and FEB, which stimulated the production of fouling-causing SMP (induced the composition change) by FCB and consequently resulted in enhancement of membrane fouling.

*Mesorhizobium* and *Thermomonas* have been frequently detected in MBRs^40–42^. They were also found in both mixed liquor (approximately 0.004% and 1.6% of total reads analyzed by next-generation sequencing, respectively) and gel layer on fouled membrane (0.01% and 2.9% of the total reads, respectively) in the MBR where the bacterial strains were isolated^8^. *Mesorhizobium* is capable of producing AHL^31^. *M. tianshanense* possess MtrI and MtrR, which are homolog of LuxR and LuxI family proteins and act as a QS regulatory system^32^. In this genus, QS system has been linked to the regulation of nodulation efficiency, growth rate, and exopolysaccharide production, and nitrogen fixation^43, 44^. In contrast, there is limited information on the AHL-mediated QS of *Thermomonas* although it was reported that the growth was likely promoted by AHL^45^.

In addition to AHL-mediated QS systems, existence of quorum-quenching (QQ) systems that block the QS systems in MBRs has been reported^15, 45, 46^. Therefore, complex microbial interactions among QS bacteria, QQ bacteria, FCB, and FEB must be studied in the future for better understanding of membrane fouling in MBRs. In addition, possible combinations of QQ and other approaches such as chlorination^47^, enrichment of fouling-reducing bacteria (i.e. *Bacillus* and *Chloroflexi*)^48, 49^, and anodic respiration^24^ should be investigated for efficient membrane fouling mitigation.

Since microfiltration (MF) membrane with pore size of 0.2 μm was used in this study, a portion of SMP and AHLs might pass through the membrane instead of rejected by and remained on the membrane. Therefore, the membrane fouling potentials measured in this study would not represent correctly the fouling potentials of the isolated bacterial strains.

In conclusion, the effect of co-culturing bacterial strains isolated from a pilot-scale MBR on membrane fouling were investigated in the present study. It was found that FCB, especially S26 (closely related to *Mesorhizobium ciceri* (98.4%)), induced severe membrane fouling when co-cultured with non-FCB and activated sludge, suggesting that FCB were mainly responsible for membrane fouling in the pilot-scale MBRs treating domestic wastewater. In particular, the fouling potential of S26 was increased 26.8 times when cultivated with S22 (closely related to *Thermomonas fusca* (98.5%)). The mechanism enhancing membrane fouling in this co-culture was further investigated in detail. As a result, co-culturing S22 and S26 induced the increase in C8-HSL production and thereby stimulated the production of fouling-causing SMP (induced the composition change) by S26, which consequently resulted in enhancement of membrane fouling. Taken together, AHL-mediated QS system was involved in SMP production and membrane fouling, which could not be revealed by single-culture studies.

## Electronic supplementary material

Supplemental Information
