# Supplementary material for: Membrane fouling induced by AHL-mediated soluble microbial product (SMP) formation by fouling-causing bacteria co-cultured with fouling-enhancing bacteria
Source: Sci Rep. 2017 Aug 16;7:8482. doi: 10.1038/s41598-017-09023-5 (PMC5559553; doi:10.1038/s41598-017-09023-5)
Supplement: Supplementary file 1 — Supplemental Information [file 41598_2017_9023_MOESM1_ESM.pdf]

Supplemental Information

Membrane fouling induced by AHL-mediated soluble microbial product (SMP) formation by fouling-causing bacteria co-cultured with fouling-enhancing bacteria

So Ishizaki[1], Ryoichi Sugiyama[1], and Satoshi Okabe[1]*

[1]Division of Environmental Engineering, Faculty of Engineering, Hokkaido University, North 13, West 8, Kita-ku, Sapporo, Hokkaido 060-8628, Japan

*Corresponding Author:

sokabe@eng.hokudai.ac.jp

Tel: +81-11-706-6266

Fax: +81-11-706-6266

Supporting information

5 pages

3 figures

2 tables

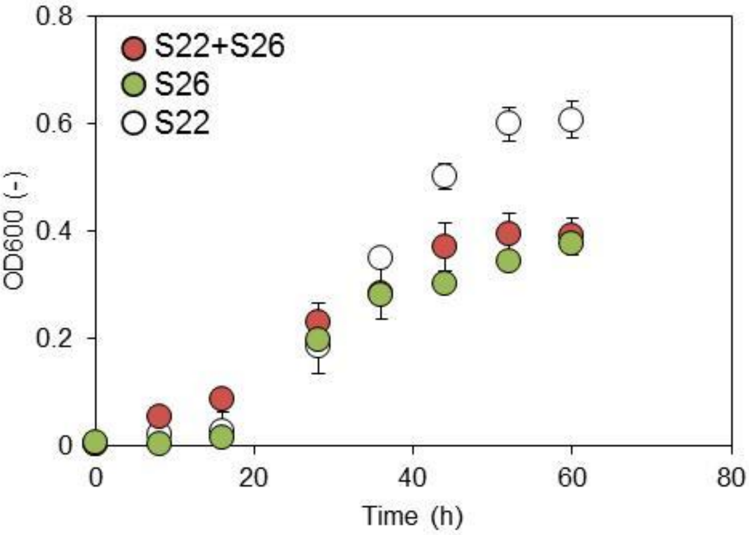

Fig. S1 Growth curves of single-culture S22, S26, and co-culture of S22 and S26.

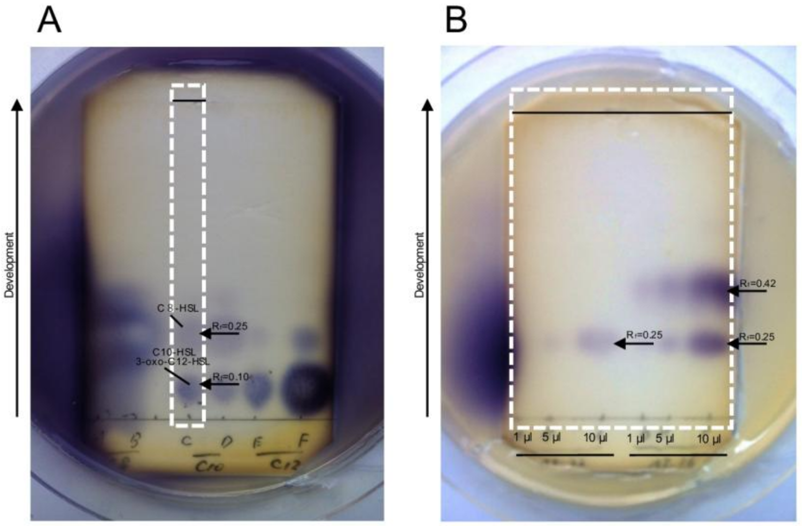

Fig. S2 Full-size images of TLC assay shown in Fig. 4A. (A) standard AHL and (B) AHL produced by S22 and S26 were blotted on TLC plates, respectively. The areas surrounded by white dotted lines were depicted in Fig. 4A

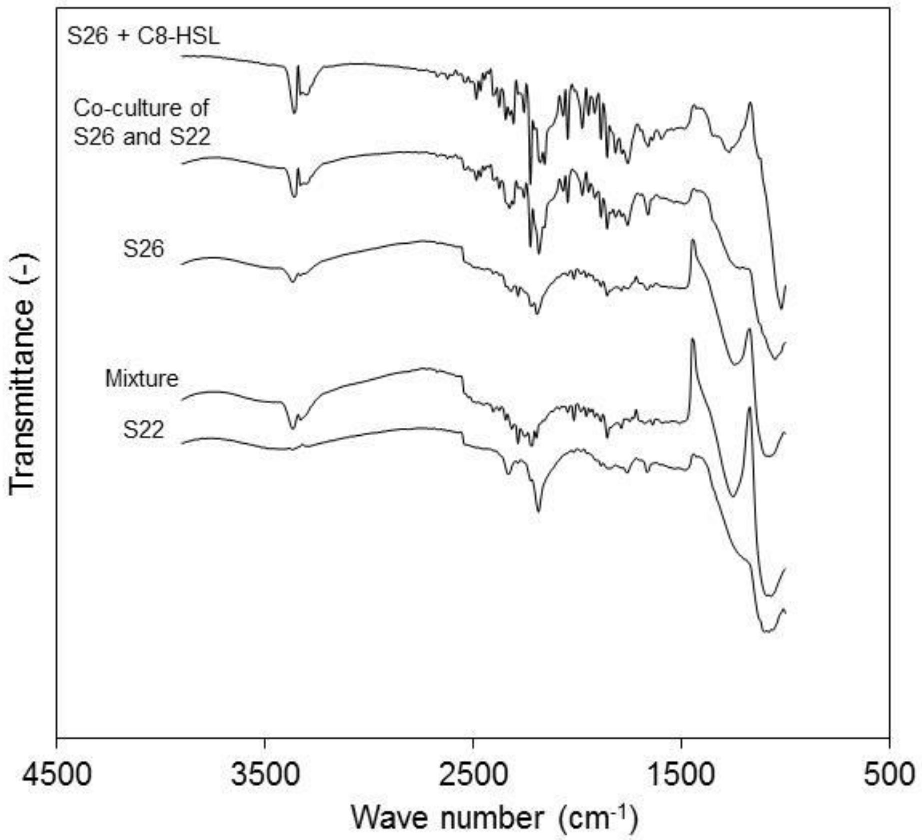

Fig. S3 FTIR spectra of SMP produced single-culture S22, S26, mixture of S22 and S26, co-culture of S22 and S26, and S26 cultured with C8-HSL.

Table S1 Summary of fouling potential of all the combination of isolated strains.

		Strain□												
	Strain	S01	S05	S09	S12	S14	S15	S18	S20	S22	S26	S31	S32	S40
Strain cultivated with□	S01	4.8E+10	6.1E+10	6.0E+10	4.9E+10	5.5E+10	6.2E+10	4.4E+10	4.3E+10	4.6E+10	8.2E+10	4.6E+10	1.2E+11	4.5E+10
	S05	6.1E+10	3.8E+11	1.9E+11	5.9E+11	1.3E+11	4.3E+11	1.9E+11	2.8E+11	4.1E+11	2.1E+11	5.4E+11	2.4E+11	5.7E+11
	S09	6.0E+10	1.9E+11	6.7E+10	4.4E+10	1.1E+11	7.5E+10	8.1E+10	6.1E+10	6.0E+10	1.8E+11	1.0E+11	3.0E+11	6.2E+10
	S12	4.9E+10	5.9E+11	4.4E+10	4.2E+10	5.1E+10	5.6E+10	3.5E+10	3.9E+10	4.4E+10	6.2E+10	4.6E+10	4.2E+10	4.8E+10
	S14	5.5E+10	1.3E+11	1.1E+11	5.1E+10	5.2E+10	5.6E+10	5.0E+10	4.1E+10	4.2E+10	5.8E+10	4.6E+10	4.8E+10	6.2E+10
	S15	6.2E+10	4.3E+11	7.5E+10	5.6E+10	5.6E+10	3.6E+10	3.7E+10	3.5E+10	3.8E+10	1.2E+11	3.8E+10	1.7E+11	5.5E+10
	S18	4.4E+10	1.9E+11	8.1E+10	3.5E+10	5.0E+10	3.7E+10	7.7E+10	8.6E+10	9.1E+10	6.1E+11	7.2E+10	2.5E+11	1.0E+11
	S20	4.3E+10	2.8E+11	6.1E+10	3.9E+10	4.1E+10	3.5E+10	8.6E+10	5.7E+10	4.6E+10	4.3E+11	5.3E+10	9.4E+10	3.6E+11
	S22	4.6E+10	4.1E+11	6.0E+10	4.4E+10	4.2E+10	3.8E+10	9.1E+10	4.6E+10	4.6E+10	1.1E+13	1.4E+11	5.3E+11	8.0E+10
	S26	8.2E+10	2.1E+11	1.8E+11	6.2E+10	5.8E+10	1.2E+11	6.1E+11	4.3E+11	1.1E+13	4.3E+11	3.3E+12	1.1E+12	1.9E+12
	S31	4.6E+10	5.4E+11	1.0E+11	4.6E+10	4.6E+10	3.8E+10	7.2E+10	5.3E+10	1.4E+11	3.3E+12	4.3E+10	1.1E+12	1.0E+11
	S32	1.2E+11	2.4E+11	3.0E+11	4.2E+10	4.8E+10	1.7E+11	2.5E+11	9.4E+10	5.3E+11	1.1E+12	1.1E+12	6.6E+11	1.2E+11
	S40	4.5E+10	5.7E+11	6.2E+10	4.8E+10	6.2E+10	5.5E+10	1.0E+11	3.6E+11	8.0E+10	1.9E+12	1.0E+11	1.2E+11	4.4E+10

Table S2 Summary of the value of OD600 of all the combination of isolated strains.

Strain□ (columns); Strain cultivated with□ (rows)

Strain	S01	S05	S09	S12	S14	S15	S18	S20	S22	S26	S31	S32	S40
S01	2.569	2.583	2.726	2.543	2.562	2.666	2.617	2.559	2.627	2.705	2.450	2.524	2.640
S05	2.583	2.005	1.728	1.696	0.741	1.064	1.360	2.354	2.375	2.436	1.254	1.145	0.699
S09	2.726	1.728	1.492	1.204	1.470	0.797	1.528	2.331	2.313	2.306	1.591	1.152	1.391
S12	2.543	1.696	1.204	0.354	1.482	0.375	2.243	2.354	2.328	2.382	1.384	0.526	0.376
S14	2.562	0.741	1.470	1.482	0.914	0.734	1.218	2.253	2.525	2.443	1.325	0.844	0.680
S15	2.666	1.064	0.797	0.375	0.734	0.387	1.070	2.664	2.221	2.047	1.100	0.503	0.700
S18	2.617	1.360	1.528	2.243	1.218	1.070	0.545	2.690	2.435	2.378	1.359	0.787	1.009
S20	2.559	2.354	2.331	2.354	2.253	2.664	2.690	2.271	2.445	2.803	1.657	2.248	1.400
S22	2.627	2.375	2.313	2.328	2.525	2.221	2.435	2.445	2.270	2.450	2.038	1.937	2.317
S26	2.705	2.436	2.306	2.382	2.443	2.047	2.378	2.803	2.450	1.426	2.127	2.168	1.145
S31	2.450	1.254	1.591	1.384	1.325	1.100	1.359	1.657	2.038	2.127	1.378	1.479	1.296
S32	2.524	1.145	1.152	0.526	0.844	0.503	0.787	2.248	1.937	2.168	1.479	0.370	1.593
S40	2.640	0.699	1.391	0.376	0.680	0.700	1.009	1.400	2.317	1.145	1.296	1.593	1.369
